# Mesenchymal stem cells transplanted into spinal cord injury adopt immune cell-like characteristics

**DOI:** 10.1186/s13287-019-1218-9

**Published:** 2019-04-03

**Authors:** Ramil Hakim, Ruxandra Covacu, Vasilios Zachariadis, Arvid Frostell, Sreenivasa Raghavan Sankavaram, Lou Brundin, Mikael Svensson

**Affiliations:** 1 Department of Neurology, Karolinska University Hospital, 17176 Stockholm, Sweden; 2 Center for Molecular Medicine, Karolinska Institutet, 17176 Stockholm, Sweden; 3 Department of Clinical Neuroscience, Karolinska Institutet, 17176 Stockholm, Sweden; 4 Department of Oncology and Pathology, Karolinska Institutet, 17176 Stockholm, Sweden; 5 BioClinicum, Karolinska University Hospital, Solnavägen 30, Solna, 171 64 Stockholm, Sweden; 6 Department of Neurosurgery, Karolinska University Hospital, 17176 Stockholm, Sweden

**Keywords:** Mesenchymal stem cells, Spinal cord injury, Cellular response, Transcriptional changes, Phenotypes, Differentiation, Proliferation, Graft survival

## Abstract

**Background:**

Mesenchymal stem cells (MSCs) and their cellular response to various stimuli have been characterized in great detail in culture conditions. In contrast, the cellular response of MSCs in an in vivo setting is still uncharted territory. In this study, we investigated the cellular response of MSCs following transplantation into spinal cord injury (SCI).

**Methods:**

Mouse bone marrow-derived MSCs were transplanted 24 h following severe contusion SCI in mice. As controls, MSCs transplanted to the uninjured spinal cord and non-transplanted MSCs were used. At 7 days post transplantation, the MSCs were isolated from the SCI, and their global transcriptional changes, survival, differentiation, proliferation, apoptosis, and phenotypes were investigated using RNA sequencing, immunohistochemistry, and flow cytometry.

**Results:**

MSCs transplanted into SCI downregulated genes related to cell-cycle regulation/progression, DNA metabolic/biosynthetic process, and DNA repair and upregulated genes related to immune system response, cytokine production/response, response to stress/stimuli, signal transduction and signaling pathways, apoptosis, and phagocytosis/endocytosis. MSCs maintained their surface expression of Sca1 and CD29 but upregulated expression of CD45 following transplantation. Transplanted MSCs maintained their surface expression of MHC-I but upregulated surface expression of MHC-II. Transplanted MSCs survived and proliferated to a low extent, did not express Caspase-3, and did not differentiate into neurons or astrocytes.

**Conclusion:**

MSCs transplanted into SCI upregulate expression of CD45 and MHC-II and expression of genes related to cytokine production, phagocytosis/endocytosis, and immune cells/response and thereby adopt immune cell-like characteristics within the recipient.

**Electronic supplementary material:**

The online version of this article (10.1186/s13287-019-1218-9) contains supplementary material, which is available to authorized users.

## Background

Traumatic spinal cord injury (SCI) leaves an affected individual with reduced motor, sensory, and autonomic functions as well as reduced quality of life. Mesenchymal stem cells (MSCs) are fibroblast-like cells that adhere to plastic, grow in colonies, self-renew, and differentiate along the mesodermal lineage. MSCs transplanted following SCI in rodents reduce glial scar formation, cystic cavity size, and lesion size and enhance angiogenesis, tissue sparing, axonal regeneration, and re-myelination. They also alternatively activate macrophages and reduce inflammation. These beneficial modulations have been correlated with improved recovery in hind limb motor function [1–8]. It has been suggested that transplanted MSCs differentiate along the ectodermal lineage [4, 6, 9], but others have suggested different mechanisms [2, 10, 11]. Transplanted MSCs have demonstrated the capacity to enhance the expression of neurotrophic and growth factors in the recipient [2, 4, 7]. Both the differentiation and the elevation of neurotrophic and growth factors have been proposed as possible explanations for the enhanced recovery of hind limb motor function [2, 7, 9, 12, 13]. In culture conditions, MSCs release extracellular vesicles (MSC-EVs) containing proteins, trophic factors, cytokines [14, 15], mRNA [14, 16], and microRNA [14, 17, 18] through which they modify neighboring cells and mediate neuroprotective and immunomodulatory effects [19–21]. The regenerative potential of MSC-EVs seems to depend on the culture conditions [22, 23]. For the time being, the release of MSC-EVs is thought to be the main mechanism of action of MSCs.

The cellular response of MSCs in culture and the effect of MSC transplantation following SCI have been reported repeatedly. However, the SCI environment is vastly more complex than what can be simulated in culture conditions, and observations made concerning the cellular response of MSCs in culture conditions cannot be assumed to valid in an in vivo environment. Thus, in this study, we investigated the cellular response of transplanted MSCs following SCI with regard to their transcriptional changes, phenotypic profile, proliferation, apoptosis, and differentiation. Understanding the cellular response and especially the mechanism of action of MSCs allows for modification, enhancement, or replacement of MSCs, which could ultimately be transformed into a cell-free therapy that maximizes the patient’s recovery following SCI.

## Methods

### Mice

Wild-type female mice (C57BL/6J, 10–12 weeks, 18–20 g) were purchased from Scanbur (Stockholm, Sweden). Animals were kept at 20 °C ± 1 °C in a room equipped with a 12-h:12-h light/dark cycle switch. Animal care and experiments were approved by the local ethical committee (Stockholm, Sweden) and maintained according to permits (N196/15, N12317-2017, N38/16) and local guidelines at Karolinska Institutet.

### Contusion spinal cord injury

Animals were anesthetized using 0.5 mg/kg medetomidine i.p. (Domitor® vet., Orion Pharma Animal Health, 1 mg/ml) and 75 mg/kg ketamine i.p. (Ketador vet., Salfarm Scandinavia, 100 mg/ml) in combination with 0.05 mg/kg buprenorphine s.c. (Temgesic®, Indivior 0.3 mg/ml) and 5 mg/kg carprofen s.c. (Rimadyl® vet. Orion Pharma Animal Health, 50 mg/ml). Animals were given 0.1 ml normal saline s.c., weighed, and shaved on the back. The skin was incised, and the paravertebral muscles separated. The vertebrae at Th10 were stabilized using bilateral fixators in a stereotaxic frame (Model 900 & 900-c, Kopf®). Using a surgical drill (Anspach®, EMAX® 2), the dorsal part of the vertebrae was removed. The dura mater was cut open. Using the infinite horizon impactor (Infinite Horizon, IH-0400), a severe contusion spinal cord injury (SCI, 75 kdyn) was induced at Th10. When no hemorrhage was visible in the injury area, bupivacaine (Marcain®, Aspen Nordic, 2.5 mg/ml) was injected into the surrounding tissue, and the skin was sutured (Vicryl, 4.0). Buprenorphine 0.05 mg/kg was administrated twice daily and carprofen 5 mg/kg once daily for a total of 3 days post-surgery. Mice were weighed weekly, and 25% weight loss was deemed the humane endpoint. The urine bladders were manually compressed until recovery of reflexive bladder emptying.

### Establishment of mesenchymal stem cells

Bone marrow mouse mesenchymal stem cells (MSCs) were established from 4–6-week-old male C57BL/6J mice using methods described elsewhere [24]. Briefly, animals were sacrificed, and the tibia and femur from both legs were harvested. The epiphysis of the tibias and femurs were cut open, and the bone marrow was extruded by flushing with pre-warmed basal medium (89% α-MEM (Gibco®, 22561054), 1% Pen-Strep (Gibco®, 10.000 U/ml, 15140122), and 10% fetal bovine serum (Gibco®, 10082147)). The bone marrow was dissociated by trituration and filtered through a 70-μm cell strainer (Corning, Inc., 352350). Bone marrow cells were re-suspended in basal medium and plated at a density of 1.45 × 10^6^ cells/cm^2^ in 100-mm tissue culture-treated dishes (150350, Nunc™). At 8–10 days post initial plating, the plastic adherent bone marrow cells were harvested using 0.5% trypsin (Gibco™, 15400054) for immune depletion. MSCs were depleted of CD34-positive (eBioscience, 13-0341-85), CD45-positive (BD, 553078), and CD11b-positive (BD, 553309) cells. Immune-depleted MSCs were characterized in terms of differentiation potential, phenotypic profile, colony-forming potential, and growth dynamics.

### Transfection of mesenchymal stem cells

A modified enhanced episomal vector system (EEV; CAT#EEV600A-1, System Biosciences) was used to mark the MSC genetically with mCherry. A construct containing the mCherry cDNA linked to the cDNA of the HBEGF/DTR [25, 26] via a 2a self-cleaving peptide linker was directionally cloned into the multiple cloning sites of the vector, downstream of the CAG promoter. Prior to transfection, the plasmid was cleared from endotoxins by using the endo-free kit (Qiagen, 12362). At 96 h prior to transplantation, MSCs were plated at a density of 10e3 cells/cm^2^ in tissue culture-treated six-well plates (Corning™, 3516). At 48 h prior to transplantation, the MSCs were transfected (2.5 μl cDNA/well) using Lipofectamine™ 3000 (Invitrogen, L3000001). The transfection efficiency was 40–50% (Additional file 1: Figure S1A-C).

### Transplantation of mesenchymal stem cells

Prior to transplantation (24 h post SCI, 48 h post transfection), the fluorescence (mCherry) of the MSCs was confirmed (Zeiss, Axiovert 200). The MSCs were harvested using 0.5% trypsin, washed and collected in 0.2-ml Eppendorf tubes, and kept at 4 °C until transplantation. Animals were anesthetized using the same principles as during SCI induction, the sutures were opened, and the spinal cord was exposed. Using a glass capillary needle (WPI, 1B150F-6) attached to a 10-μl syringe (Hamilton®, 80330), ~ 0.5 × 10^6^ MSCs were injected into the SCI epicenter under microscopic visualization. The skin was sutured (Vicryl, 4.0), and post-operative procedures as described for SCI were implemented.

### Sacrifice and tissue harvesting

At 7 days post transplantation, animals were euthanized using pentobarbitalnatrium. For histological evaluation, animals were sacrificed at 7 and 14 days post transplantation. Animals were transcardially perfused with 1×PBS using a peristaltic pump (Watson Marlow, 120S). Spinal cords intended for evaluation using flow cytometry of fluorescence-activated cell sorting (FACS) were dissected and stored in 1×PBS on ice. Animals intended for histological evaluation were further perfused with paraformaldehyde (PFA, 4%). These spinal cords were dissected and post-fixated in PFA overnight.

### Isolation of transplanted mesenchymal stem cells from the spinal cord

Spinal cords were dissociated using trituration in L-15 medium (Thermo Fisher, 11415064) containing 10 U/ml papain (Worthington, L5003126). Two hundred units per milliliter DNAse (Sigma, D7291) was added during the dissociation. Papain was inactivated using 1% bovine serum albumin (BSA; Gibco®, 15260-037) in 1×DPBS. Myelin was removed using a 30% Percoll (Sigma, P1644) gradient and centrifugation at 750×*g* for 10 min at 4 °C with slow brake. The pellet was re-suspended in FACS buffer (1% BSA, 2 mM EDTA (Gibco®, 15575-038), 25 mM HEPES (Sigma, H0887)) and filtered through a pre-wet 100-μm cell strainer (Corning, 431752) followed by a pre-wet 40-μm strainer (Corning, 431750). mCherry+MSCs (Additional file 2: Figure S2A-R) were isolated from the dissociated spinal cord using FACS (BD Influx™). Sorted cells (28,000 ± 14,000 MSCs) were collected in FACS buffer, centrifuged at 300×*g* for 5 min, and re-suspended in 1 ml Trizol reagent (Thermo Fisher, 15596026), incubated for 5 min, vortexed, frozen on dry ice, and stored at −70 °C until downstream processing.

### Extraction of RNA from isolated mesenchymal stem cells

RNA from isolated MSCs was isolated using Trizol (manufacturer’s protocol). Contaminating genomic DNA was removed during the RNA isolation by on-column digestion with DNAse (DNAse I Qiagen, 79254). RNA clean-up was conducted using the RNeasy micro kit (Qiagen, 74004). RNA was stored at − 70 °C until sequencing.

### Analysis of global transcriptional changes in mesenchymal stem cells

Sequencing libraries were prepared using the SMARTer Stranded Total RNA-Seq Kit - Pico Input Mammalian kit (Clontech). Libraries were sequenced 2 × 125 bp in two lanes using the HiSeq2500 system and v4 sequencing chemistry (Illumina Inc.) to a combined total of at least 15.7 × 10^6^ reads/sample. TrimGalore (Babraham Bioinformatics) was used for the removal of adapter sequences and low-quality regions. The splice-aware aligner STAR was used for aligning remaining pair-end reads to the mouse genome (build GRCm38). FeatureCounts and Ensembl annotation (release 81) were used for summarization of read counts over genes. Annotation and data analysis were conducted in R (version 3.5.1) using packages limma and edgeR with annotations from Mus.musculus (https://www.bioconductor.org/), GEO accession number: GSE125176.

#### Functional analysis

Significantly differentially expressed genes (FDR < 0.01, LogFC = 1) for each contrast were analyzed using over-representation enrichment analysis (ORA) and network topology-based analysis (NTA) using WEB-based Gene SeT AnaLysis Toolkit (WebGestalt) implemented with R package “WebGestaltR.” Up- and downregulated genes in each contrast were analyzed separately. In ORA, the Gene Ontology (GO) terms related to biological process (BP), molecular function (MF), and cellular component (CC) were investigated. Furthermore, in ORA, pathways were investigated using Kyoto Encyclopedia of Genes and Genomes (KEGG) terms. In NTA, both network retrieval and prioritization (NRP) and network expansion (NE) were used for network construction. All terms (and related genes) which fulfilled FDR < 0.01 were exported for each method (BP, MF, CC, KEGG, NRP, NE), contrast and direction. Each term was then manually categorized into more general categories for enhanced interpretation. For each contrast and category, the median FDR was calculated. Competitive gene set testing accounting for inter-gene correlation was performed on all unique genes in each category for each contrast. A category was deemed significant if median FDR for GO/KEGG terms and exact FDR for competitive gene set testing were both < 0.05 for that specific category. The categories were ordered based on the number of GO/KEGG terms that were detected for the specific category. Gene set enrichment analysis (GSEA) was conducted using Molecular Signatures Database (MSigDB, v6.2) using collections: hallmark gene sets, curated gene sets (C2), and immunologic gene sets (C7).

### Flow cytometry

Spinal cords containing transplanted MSCs were dissociated using papain (above). Cells were blocked in Mouse Fc Block (BD, 553141) for 5 min and stained using pre-conjugated antibodies (Table 1) in 100-μl FACS buffer on ice for 30 min. Following the wash, the cells were re-suspended in 250 μl FACS buffer. Non-transplanted MSCs were harvested (above) from culture 48 h post transfection and stained in the same fashion. Flow cytometry was conducted using a BD LSRFortessa™ cell analyzer and data analyzed in Kaluza Analysis Software (Beckman Coulter).

**Table 1:** Primary, secondary, and pre-conjugated antibodies

Antibody type	Host	Target	Fluorochrome	Manufacturer (ID)	Dilution
Primary	Mouse	NeuN	NA	Merck Millipore (MAB377)	1:1000
	Mouse	TuJ1	NA	Merck Millipore (MAB1637)	1:100
	Rabbit	GFAP	NA	Dako (Z0334)	1:1000
	Rat	MHC-II	NA	Abcam (ab25333)	1:200
	Rabbit	Caspase-3	NA	Abcam (ab13847)	1:100
Secondary	Goat	Mouse	Alexa 488	Life Technologies (A11001)	1:500
	Goat	Rabbit	Alexa 488	Life Technologies (A11008)	1:400
Pre-conjugated	NA	CD29	APC	eBioscience (17-0291-80)	1 μg per 1 × 10^6^ cells
	NA	Sca1	FITC	BD (561077)	
	NA	CD45	v450	BD (560697)	
	NA	MHC-I	FITC	BioLegend (125508)	
	NA	MHC-II	FITC	BioLegend (107606)	

### Immunohistochemistry

Post-fixed spinal cords were cryo-protected in 15% and 30% sucrose (Sigma, S9378) in 1×PBS. Spinal cords were mounted in cryomolds (Tissue-Tek® Cryomold®, 420572) using compound (Tissue-Tek® O.C.T.™) and rapidly frozen to − 60 °C. Twenty-micrometer coronal sections were produced using a cryostat (Leica, CM1850, − 22 °C) and mounted on slides (VWR, SuperFrost® Plus, 48311-703). Sections were thawed, rehydrated in 1×PBS, blocked for 2 h at RT in blocking solution (0.3% Triton X-100 (Sigma, 93443), 5% normal goat serum (Serotec, 301104, 1×PBS and 0.01% sodium azide (Sigma, S-2002)). Primary antibody (Table 1) was added, and sections were incubated at 4 °C for 24 h. Sections were rinsed in 1×PBS followed by incubation in secondary antibody (Table 1) at RT for 1 h. Sections were incubated at RT for 20 min with nucleic acid stain (Hoechst 33258, Invitrogen™ H3569). Prior to confocal microscopy, the slides were rinsed and mounted using Mowiol (Sigma, 81381) and a cover slip (Marienfeld, 010243).

### Immunocytochemistry

FACS isolated MSCs were collected at 500×*g* for 5 min at 4 °C and re-suspended in basal medium. Cells were plated at a density of 20.000 cells/cm^2^ on tissue culture-coated slides (Nunc™ Lab-Tek® Chamber Slide™, 177402) in 0.3-ml basal medium. Following 3 days of culture, the MSCs were washed and fixated using 2% PFA for 30 min. MSCs were stained using the procedure described for frozen sections and imaged using confocal microscopy.

### Proliferation assay

MSCs in culture were exposed to 1:1000 5-ethynyl-2′-deoxyuridine (EdU; Thermo Fisher, A10044) for 24 h prior to assessment of proliferation. Proliferation of transplanted MSCs was assessed by administration of 0.75 mg/ml EdU in the drinking water of the mice. Drinking water containing EdU was offered to the animals from transplantation to sacrifice. The water was supplemented with 1% sucrose (Sigma Aldrich, S0389). Proliferation of MSCs in culture was assessed using flow cytometry (above). Proliferation of transplanted MSCs was assessed using immunohistochemistry. For both evaluations, the Click-iT™ Plus EdU Alexa Fluor™ 488/555 Imaging Kit (Thermo Fisher, C10637) was used according to the manufacturer’s instructions.

### Differentiation assay

Differentiation of transplanted MSCs was evaluated using immunohistochemistry. The co-expression of mCherry and either GFAP, NeuN, or TuJ1 (Table 1) was evaluated using confocal microscopy.

### Confocal microscopy and automatic cell quantification

Stained frozen sections and stained fixed MSCs were imaged using a confocal microscope (Zeiss LSM 880 Airyscan). For estimation of proliferation, differentiation, apoptosis, and survival of mCherry+MSCs, all cells in four random sections from every animal were imaged for analysis. Orthogonal projections using × 40 as well as overview images using × 20 for quantification were collected. The proportion of MSCs co-expressing mCherry and the specific marker was estimated using a custom built automatic cell quantification macro (https://github.com/S-B-lab/confocal_image_quantifier) implemented in Image Processing and Analysis in Java (ImageJ, 64-bit Java 1.6.0_24). For each animal and staining, the images were imported, and an image sequence was created. The macro allows the user to select one, two, or three channels for evaluation of co-localization. The macro splits the image into its three color channels. The selected color channels are multiplied, and the image is converted to binary format and water-shedded. Co-localized cells are then identified and labeled based on user-defined settings for desired size and circularity of cells. The accuracy was always evaluated using overlays. In case the macro had too low accuracy in a given image sequence, a manual cell count was done instead using the “Cell Counter” plugin in ImageJ.

### Statistical analysis

Mean with 95% confidence interval or median with range (25th and 75th percentile) was presented when appropriate. *p* values < 0.05 were considered significant. When applicable, the assumption of normality of the data was evaluated using Shapiro-Wilk’s test. The assumption of homogeneity of variances between groups was evaluated using Bartlett’s test when data was normally distributed and Fligner-Killeen’s test when data departed from normality. Depending on the fulfillment of assumptions, multiple group comparisons were evaluated using Kruskal-Wallis *H* test followed by pairwise comparisons between group levels with correction for multiple testing using Holm’s method or using one-way ANOVA followed by Tukey’s post hoc test. Independent two-group comparisons were conducted using the Wilcoxon rank-sum test or Student’s *t* test depending on the fulfillment of assumptions. Analysis was conducted, and figures were prepared in R (version 3.5.1) mainly using packages data.table and ggplot2.

## Results

### MSCs transplanted into SCI downregulate genes related to cell-cycle and DNA metabolic/biosynthetic processes and upregulate genes related to immune system response, cytokine production, and phagocytosis/endocytosis

**Fig. 1:**
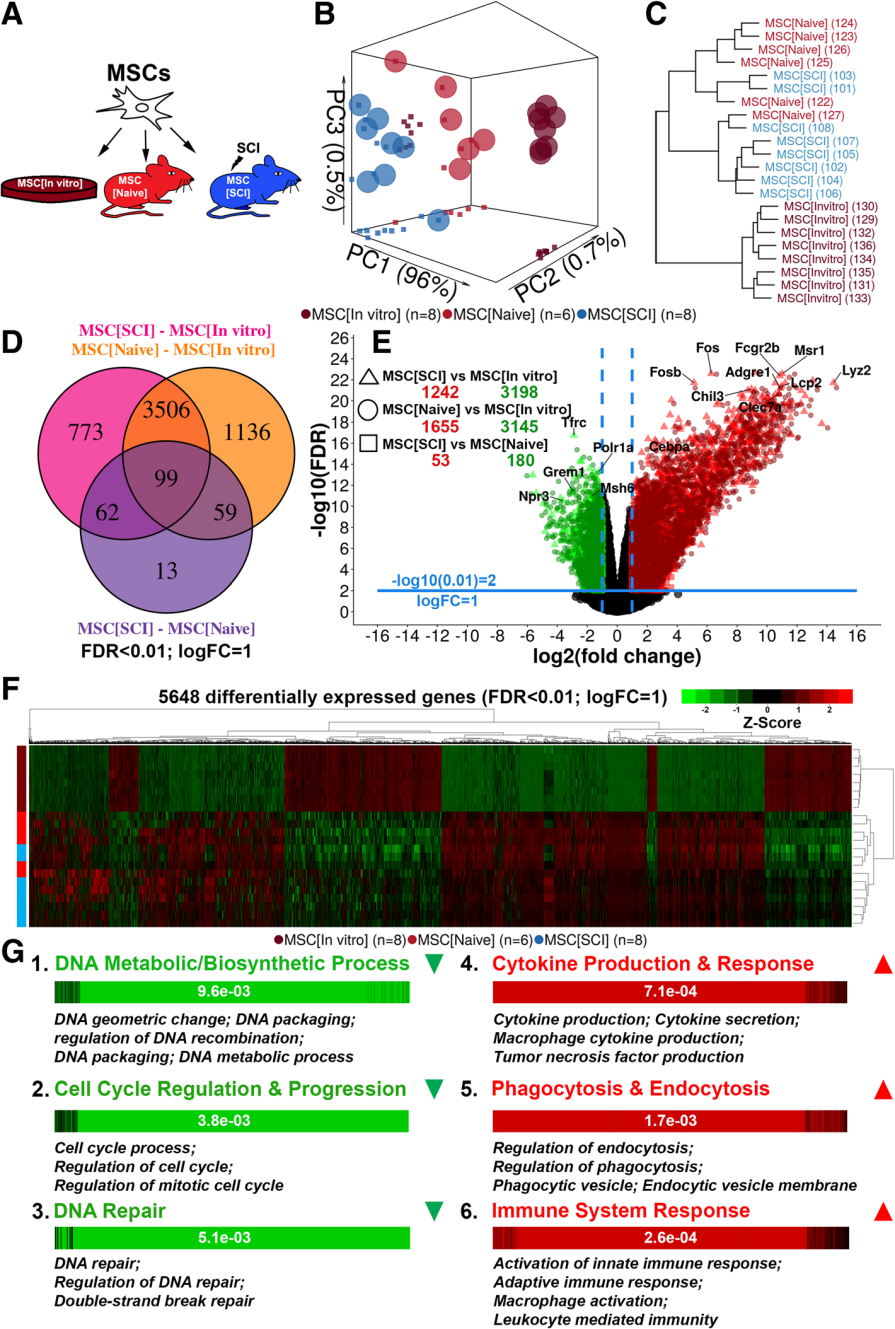
Global transcriptional changes in MSCs. **a** Experimental design. Transplanted MSCs were evaluated 7 days following transplantation. **b** Principal component analysis using the 500 genes with the highest inter-sample variance. Spheres represent biological replicates in 3D, while squared dots represent biological replicates in 2D (on the walls of the box), comparing two components at a time. **c** Agglomerative hierarchical clustering of biological replicates presented using a dendrogram. Data is based on the value of the first and second principal component for each biological replicate. Numbers in parentheses are animal index numbers. **d** Unique number of significantly differentially expressed genes in each contrast and the number of genes shared between the contrasts. Color of text specifying the contrast corresponds to the color of the circle in the Venn diagram. **e** Differentially expressed genes presented using a volcano plot. **f** Heat map of unique significantly differentially expressed genes based on all three contrasts with row- and column-wise agglomerative hierarchical clustering. **g** Up- and downregulated categories for contrasts MSC[SCI] vs MSC[In vitro] and MSC[Naive] vs MSC[In vitro]. Barcode plots are presented with a *p* value determined using a competitive gene set test. Representative Gene Ontology (biological process) terms are presented for each category. Green color indicates a category downregulated in MSC[SCI]/MSC[Naive], while red indicates a category upregulated in MSC[SCI]/MSC[Naive] as compared to MSC[In vitro]

MSCs transfected to express mCherry were transplanted into the uninjured and injured spinal cord. At 7 days following transplantation, the MSCs were isolated using FACS. MSCs transplanted to spinal cord injury (SCI) were compared to MSCs transplanted into the uninjured spinal cord and to non-transplanted MSCs in terms of global transcriptional changes (Fig. 1a). Dimensionality reduction (Fig. 1b, Additional file 3: Figure S3A) and clustering (Fig. 1c, Additional file 3: Figure S3B, C) revealed a distinct separation between transplanted (MSC[SCI], MSC[Naive]) and non-transplanted MSCs (MSC[In vitro]). A more modest separation was detected between MSC[SCI] and MSC[Naive]. Numerous significantly differentially expressed genes were detected in contrasts MSC[SCI] vs MSC[In vitro] and MSC[Naive] vs MSC[In vitro] but not in MSC[SCI] vs MSC[Naive] indicating similarity between transplanted MSCs but a dissimilarity between transplanted and non-transplanted MSCs (Fig. 1d, e). Heat map representation of all significantly differentially expressed genes confirmed that the largest differences were found between transplanted and non-transplanted MSCs and that transplanted MSCs upregulated rather than downregulated their gene expression (Fig. 1f). Functional analysis revealed that genes mainly related to cell-cycle regulation/progression, DNA metabolic/biosynthetic processes, and DNA repair were downregulated and genes related to immune system response, cytokine production/response, response to stress/stimuli, signal transduction and signaling pathways, apoptosis, and phagocytosis/endocytosis were upregulated in transplanted MSCs as compared to non-transplanted MSCs (Fig. 1g, Table 2). Immune system response was characterized by genes related to both the innate and the adaptive immune system as indicated by the GO terms: *activation of innate immune response*, *adaptive immune response*, *activation of immune response*, and *immune system process* but also the activation of macrophages (*macrophage activation*). Interestingly, the cytokine production was related to cytokines commonly produced by macrophages and were mainly pro-inflammatory as indicated by the GO terms: *interleukin-6 production*, *tumor necrosis factor production*, *interleukin-1 production*, *interferon-gamma production*, *interleukin-12 production*, *chemokine production*, *interleukin-8 production*, *interleukin-10 production*, *interleukin-2 production*, and *positive regulation of interleukin-17 production.* Thus, the MSCs seem to adopt a secretory profile similar to classically activated macrophages. The genes related to signal transduction and signaling pathways were related to a variety of pathways as indicated by the GO terms: *regulation of kinase activity*, *regulation of MAPK cascade*, *Ras protein signal transduction*, *Fc receptor signaling pathway*, *regulation of small GTPase mediated signal transduction*, and *Jak-STAT signaling pathway.* Taken together, transplanted MSCs suppress genes related to cell-cycle activity and DNA processes/repair but upregulate genes related to cytokine production/response, immune system cells, and phagocytosis/endocytosis, suggesting that transplanted MSCs adopt immune cell-like characteristics.

**Table 2:** Functional analysis of significantly differentially expressed genes (FDR < 0.01; LogFC = 1)

Contrast	Direction	Category	ORA and NTA		Competitive gene set test	
			Terms (*n*)	FDR (median)	Genes (*n*)	FDR (exact)
MSC[Naive] vs MSC[In vitro]	Down	Cell-cycle regulation and progression	159	2.1e−04	276	2.3e−03
		DNA recombination/metabolic process	46	3.6e−04	412	2.9e−03
		Chromosome (condensed)	22	1.7e−06	129	5.5e−03
		DNA repair and response to stress	11	1.9e−04	64	1.9e−03
	Up	Immune system response	718	1.2e−05	940	7.6e−05
		Signaling pathway	222	3.3e−04	862	3.7e−06
		Cytokine production and response	207	7.1e−05	433	3.4e−04
		Cellular response to stress and stimuli	198	1.3e−05	1346	1.2e−05
		Metabolic process	161	2.4e−04	1345	2.3e−05
		Protein modification/assembly/transport	112	6.4e−05	1061	3.4e−05
		Ion transport and homeostasis	99	5.9e−06	420	3.4e−06
		Cell migration and chemotaxis	91	8.1e−05	461	8.9e−07
		Trans (cell) membrane transport	90	9.0e−04	372	9.8e−05
		Ubiquitination and apoptosis	83	2.0e−04	546	8.8e−05
		Signal transduction	67	9.9e−08	973	4.5e−06
		Tissue development and regeneration	63	1.3e−04	1120	8.1e−06
		Biosynthetic process	61	1.2e−03	656	8.5e−04
		Cell-cell adhesion	59	1.1e−05	625	3.7e−06
		Phagocytosis and endocytosis	43	1.7e−05	280	1.0e−03
		Catabolic process	37	1.0e−03	277	4.1e−04
		Regulation of (cell) proliferation	37	1.8e−05	524	1.7e−04
		Cell membrane activity	31	4.0e−07	791	2.9e−05
		Regulation of (cell) differentiation	28	7.4e−04	592	7.5e−07
		Synaptic function and activity	22	3.1e−03	184	7.1e−04
		Cell and tissue morphology	17	1.2e−03	496	5.6e−04
		Receptor binding	15	1.3e−03	10	6.8e−03
		Transcriptional activity	15	2.8e−03	485	1.2e−02
		I-kappaB kinase/NF-kappaB signaling	14	2.6e−04	82	2.3e−03
		Regulation of (cell) activation	12	0.0e+00	267	1.4e−03
		Regulation of cell homeostasis	11	1.5e−03	161	9.1e−04
MSC[SCI] vs MSC[In vitro]	Down	Cell-cycle regulation and progression	220	1.3e−04	253	3.8e−03
		DNA recombination/metabolic process	51	2.2e−04	370	9.6e−03
		Chromosome (condensed)	25	6.1e−08	122	8.9e−03
		DNA repair and response to stress	16	1.2e−05	76	5.1e−03
		Meiotic cell-cycle process	15	7.5e−05	49	1.1e−02
	Up	Immune system response	642	2.5e−05	836	2.6e−04
		Cellular response to stress and stimuli	176	1.7e−05	1215	5.9e−05
		Signaling pathway	173	3.5e−04	697	4.9e−05
		Cytokine production and response	157	6.5e−05	399	7.1e−04
		Metabolic process	122	7.0e−04	1126	2.0e−04
		Ion transport and homeostasis	117	4.2e−07	439	1.4e−05
		Protein modification/assembly/transport	102	3.2e−04	765	1.2e−04
		Trans (cell) membrane transport	81	4.0e−04	372	1.4e−04
		Cell migration and chemotaxis	77	1.1e−04	290	9.9e−05
		Signal transduction	73	2.8e−06	723	5.5e−05
		Tissue development and regeneration	67	2.1e−04	925	1.7e−06
		Ubiquitination and apoptosis	63	6.5e−04	472	3.3e−04
		Biosynthetic process	54	1.0e−03	558	1.1e−03
		Cell-cell adhesion	49	3.1e−05	499	1.9e−05
		Synaptic function and activity	38	2.1e−04	171	3.7e−05
		Phagocytosis and endocytosis	36	1.7e−05	242	1.7e−03
		Microtubuli and cytoskeleton	32	6.1e−04	508	2.7e−02
		Cell membrane activity	31	3.1e−06	670	1.1e−04
		Regulation of (cell) proliferation	29	2.6e−05	429	1.8e−04
		Regulation of (cell) differentiation	25	1.5e−03	506	1.3e−05
		Transcriptional activity	18	3.0e−03	356	1.6e−04
		Catabolic process	17	5.5e−03	233	1.3e−03
		Cell and tissue morphology	15	4.6e−03	275	2.3e−05
		Regulation of (cell) activation	12	5.0e−10	252	2.2e−03
		I-kappaB kinase/NF-kappaB signaling	11	5.9e−04	70	2.3e−03
		Secretion	11	2.3e−03	89	1.8e−04
		Neuronal cell activity	10	2.4e−07	366	2.7e−05
		Sensory perception process	10	1.8e−03	59	4.7e−05
MSC[SCI] vs MSC[Naive]	Up	Tissue development and regeneration	52	8.7e−04	43	6.3e−04
		Cell morphology	27	9.6e−04	36	9.6e−04

*ORA* over-representation enrichment analysis, *NTA* network topology-based analysis. Terms: unique terms identified using Gene Ontology (GO)–biological process, GO–molecular function, GO–cellular component, Kyoto Encyclopedia of Genes and Genomes (KEGG), network retrieval and prioritization (NRP), network expansion (NE). Genes: unique genes constituting the (unique) GO and KEGG terms for a specific category. Categories with a total of less than ten unique GO/KEGG terms are omitted from the table for enhanced interpretation

### Transplanted MSCs express CD29, Sca1, and CD45

**Fig. 2:**
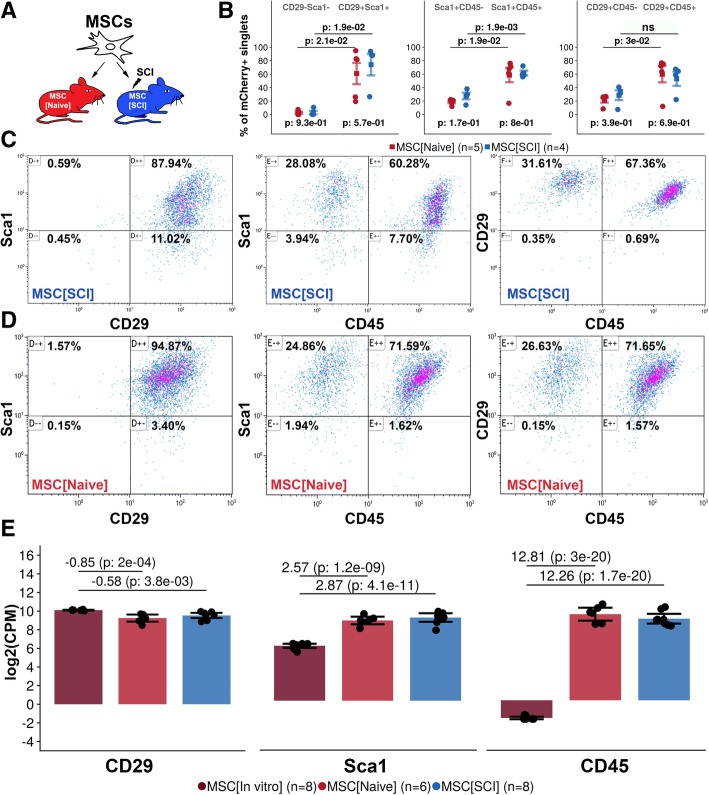
Expression of CD29, Sca1, and CD45 in transplanted MSCs. **a** Experimental design. Transplanted MSCs were evaluated 7 days following transplantation. **b** Gate statistics for each experimental group and relevant gates. Mean is surrounded by a 95% confidence interval. Each dot represents one biological replicate. Values above plot represent *p* values following independent two-group comparison within study groups between gates. Values below plot represent *p* values following independent two-group comparison within gate between study groups. **c** Representative bivariate plots of MSC[SCI]. **d** Representative bivariate plots of MSC[Naive]. **e** Log2(counts per million) for CD29, Sca1, and CD45 genes following global transcriptional analysis of MSCs. Dots represent biological replicates. Mean is surrounded by 95% confidence interval. Log2(fold change) followed by the FDR for contrasts: MSC[SCI] vs MSC[In vitro] and MSC[Naive] vs MSC[In vitro] is presented in the plot

Phenotypic characterization of positive and negative surface markers of MSCs was performed at 7 days following transplantation (Fig. 2a). A majority of non-transplanted MSCs expressed CD29 and Sca1 (Additional file 4: Figure S4C3, C4, C6). Transplanted MSCs maintained expression of CD29 and Sca1 in both injured (88.2%, CI 87.9–88.5%) (Fig. 2b, c) and uninjured (90.9%, CI 87.9–93.9%) spinal cord (*p* < 0.05) (Fig. 2b, d). Transplanted MSCs had a similar but slightly lower gene expression of CD29 (SCI − 0.58, naive − 0.85) as compared to non-transplanted MSCs (*p* < 0.01) (Fig. 2e). However, transplanted MSCs upregulated their gene expression of Sca1 (SCI 2.87, naive 2.57) as compared to non-transplanted MSCs (*p* < 0.001) (Fig. 2e). MSCs did not express CD45 in culture conditions (Additional file 4: Figure S4C5, C7, C8). Following transplantation, a majority of Sca1+ MSCs expressed CD45 in the injured (59.5%, CI 58.6–60.3%) and uninjured (58.2%, CI 56.2–60.3) spinal cord (*p* < 0.05) (Fig. 2b–d). This was also true for CD29+ MSCs in both injured (66.0%, CI 64.6–67.4%) and uninjured (63.1%, CI 58.8–67.4%) spinal cord (*p* < 0.05) (Fig. 2b–d). Transplanted MSCs significantly upregulated their gene expression of CD45 (SCI 12.81, naive 12.26) as compared to non-transplanted MSCs (*p* < 0.001) (Fig. 2e). Cells expressing Cx3cr1 aggregated around the grafted MSCs but did not infiltrate the graft at 7 days after transplantation (Additional file 5: Figure S5). Taken together, MSCs transplanted into the uninjured or injured spinal cord have a surface and gene expression of Sca1 and CD29 comparable to non-transplanted MSCs but significantly upregulate surface and gene expression of CD45 as compared to non-transplanted immunodepleted MSCs.

### Transplanted MSCs express MHC-I and MHC-II

**Fig. 3:**
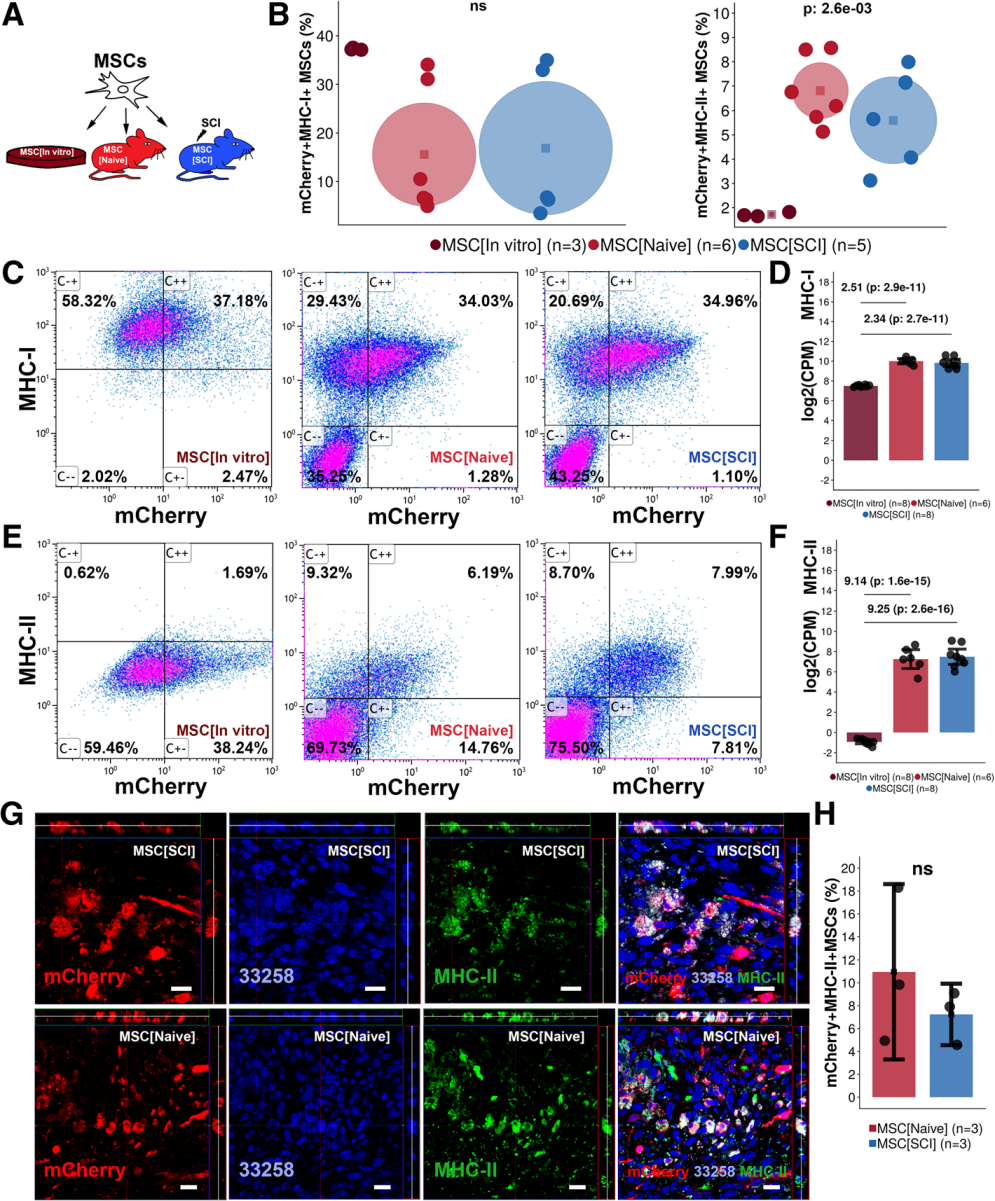
Expression of MHC-I/II in transplanted MSCs. **a** Experimental design. Transplanted MSCs were evaluated 7 days following transplantation. **b** Gate statistics for co-expression of mCherry and MHC-I/II. Mean is the center of the circle, and the diameter represents a 95% confidence interval. Each dot represents one biological replicate. Values are *p* values following a multiple group comparison test. **c** Representative bivariate plots for co-expression of MHC-I and mCherry in MSCs. **d** Log2(counts per million) for MHC-I gene following global transcriptional analysis of MSCs. Dots represent biological replicates. Mean is surrounded by a 95% confidence interval. Log2(fold change) and the FDR for contrasts: MSC[SCI] vs MSC[In vitro] and MSC[Naive] vs MSC[In vitro] are presented. **e** Representative bivariate plots for co-expression of MHC-II and mCherry in MSCs. **f** Gene expression of MHC-II presented as in **d**. **g** Surface expression of MHC-II in transplanted MSCs evaluated using immunohistochemistry. Orthogonal projections presented for each channel and the merged image. Scale bar represents 20 μm. **h** Estimation of the percentage of mCherry+MHC-II+MSCs as compared to all mCherry+MSCs in spinal cords subjected to transplantation. Dots represent biological replicates. Mean is surrounded by a 95% confidence interval. ns not significant

MSCs in culture express MHC-I but not MHC-II on the cell surface. Ninety percent of MSCs exposed to IFN-γ for 48 h in culture express MHC-II on the cell surface [27]. We hypothesized that the same type of upregulation of MHC-II on the cell surface could occur in MSCs transplanted into SCI (Fig. 3a). We confirmed that MSCs in culture express MHC-I (37.3%, CI 37.0–37.5%) but not MHC-II (1.7%, CI 1.6–1.8%) (Fig. 3b, c, e). Expression of MHC-I on the cell surface of transplanted MSCs was not significantly altered following exposure to the injured (16.9%, CI 3.1–30.6%) or uninjured (15.6%, CI 4.9–26.2%) spinal cord compared to MSCs in culture (Fig. 3b, c). Transplanted MSCs upregulated gene expression of MHC-I (SCI 2.34, naive 2.51) as compared to non-transplanted MSCs (*p* < 0.001) (Fig. 3d). In comparison, MSCs transplanted to the injured (7.6%, CI 6.7–8.4%) or uninjured (6.0%, CI 5.5–6.4%) spinal cord had a higher surface expression of MHC-II as compared to MSCs in culture (*p* < 0.01) (Fig. 3b, e). Gene expression analysis confirmed that MHC-II was significantly upregulated in transplanted MSCs (SCI 9.25, naive 9.14) as compared to non-transplanted MSCs (*p* < 0.001) (Fig. 3f). Histological evaluation of transplanted MSCs confirmed co-expression of mCherry and MHC-II in both injured and uninjured spinal cord (Fig. 3g). The percentage mCherry+MHC-II+MSCs was not significantly different between MSCs in the injured (7.2%, CI 4.5–9.9%) and uninjured spinal cord (11.0%, CI 3.3–18.6%) but comparable to findings in Fig. 3e (Fig. 3h). Taken together, MSCs transplanted into the injured or uninjured spinal cord maintain their surface and gene expression of MHC-I but upregulate surface and gene expression of MHC-II in comparison to non-transplanted MSCs.

### Transplanted MSCs survive and proliferate but do not undergo apoptosis or neural differentiation

**Fig. 4:**
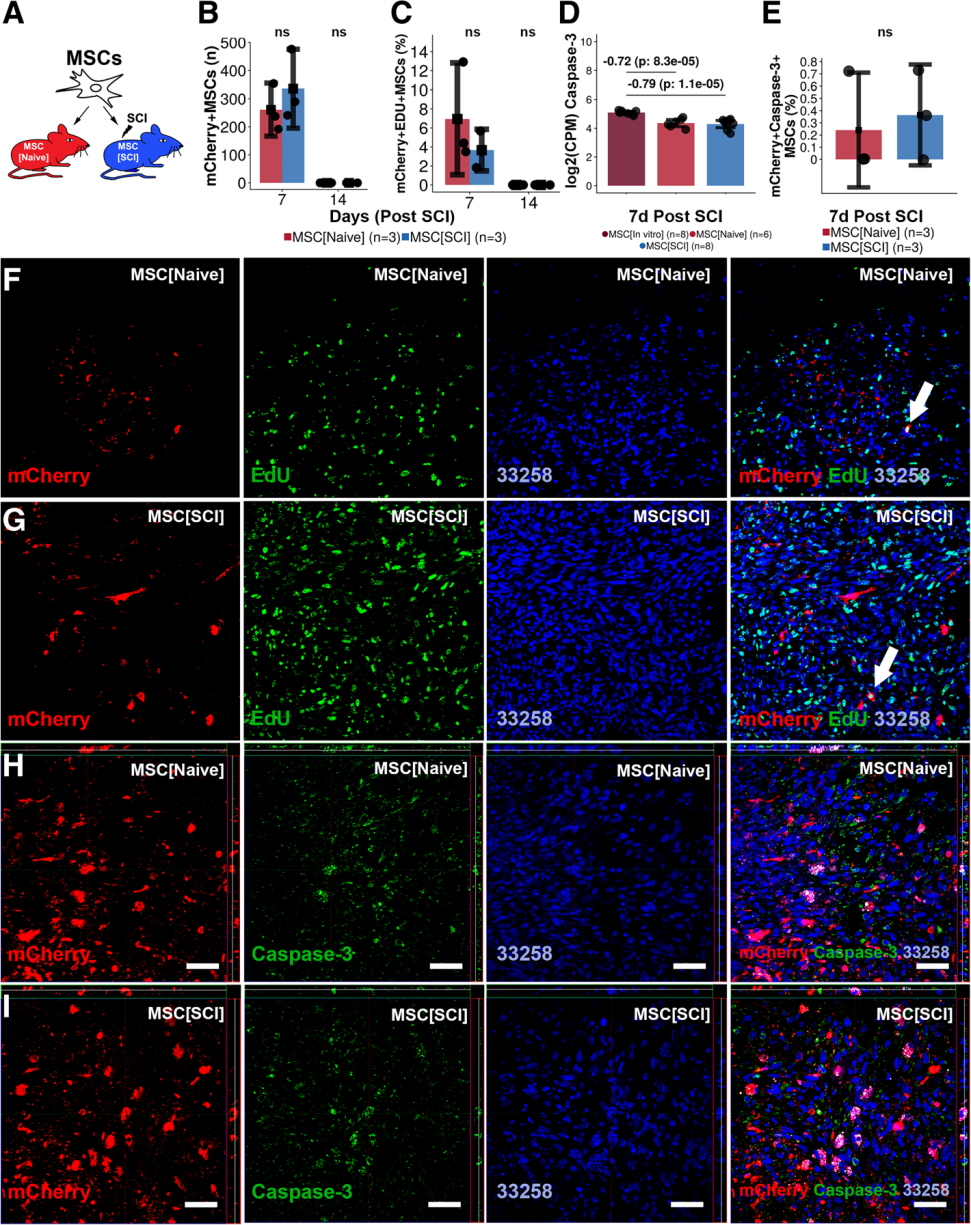
Survival, proliferation, and apoptosis of transplanted MSCs. **a** Experimental design. Transplanted MSCs were evaluated 7 and 14 days following transplantation. **b** Survival of transplanted mCherry+MSCs within the recipient. Mean surrounded by a 95% confidence interval. Each dot represents one biological replicate. Values are *p* values following independent two-group comparison between experimental groups. **c** Proliferation rate of transplanted mCherry+MSCs. Data reporting is as in **b**. **d** Log2(counts per million) for Caspase-3 gene following global transcriptional analysis of MSCs. Log2(fold change) and the FDR for contrasts: MSC[SCI] vs MSC[In vitro] and MSC[Naive] vs MSC[In vitro] are presented. Data reporting is as in **b**. **e** Estimation of the percentage of mCherry+Caspase-3+MSCs as compared to all mCherry+MSCs in spinal cords subjected to transplantation. **f** Co-expression of EdU and mCherry+MSCs in MSC[Naive] at 7 days post transplantation. **g** Equivalent in MSC[SCI]. **h** Co-expression of Caspase-3 and mCherry+MSCs in MSC[Naive] at 7 days post transplantation. **i** Co-expression of Caspase-3 and mCherry+MSCs in MSC[SCI] at 7 days post transplantation. ns not significant

**Fig. 5:**
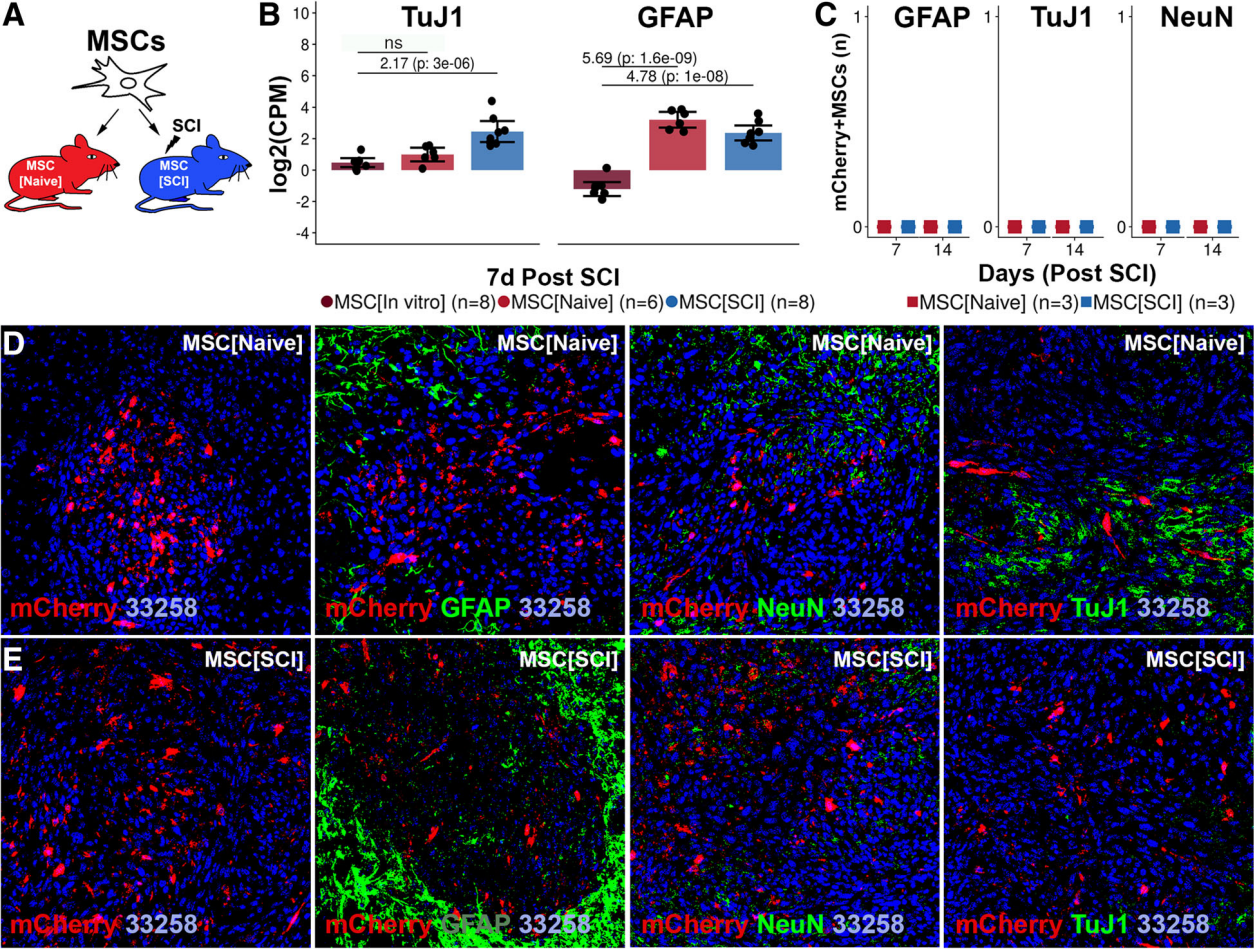
Differentiation of transplanted MSCs. **a** Experimental design. Transplanted MSCs were evaluated 7 and 14 days following transplantation. **b** Log2(counts per million) for TuJ1 and GFAP gene following the global transcriptional analysis of MSCs. NeuN gene was filtered out due to low expression. Log2(fold change) and the FDR for contrasts: MSC[SCI] vs MSC[In vitro] and MSC[Naive] vs MSC[In vitro] are presented. Mean surrounded a 95% confidence interval. Each dot represents one biological replicate. **c** Co-expression of mCherry+MSCs and GFAP, NeuN, and TuJ1, respectively. **d** Co-expression of GFAP, NeuN, TuJ1, and mCherry+MSCs in MSC[Naive] at 7 days post transplantation. **e** Equivalent to **d** for MSC[SCI]

Survival, proliferation, apoptosis, and differentiation of transplanted MSCs were assessed at 7 and 14 days post transplantation (Figs. 4a and 5a). Transplanted MSCs could be detected for 7 but not 14 days in the uninjured (261, CI 167–355) and injured (335, CI 195–476) spinal cord, respectively (Fig. 4b). The spinal cord environment did not affect MSC survival. MSCs proliferated to a low extent in both uninjured (6.9%, CI 1.1–12.8%) (Fig. 4c, f) and injured (3.7%, CI 1.5–5.9%) (Fig. 4c, g) spinal cord. Although the proliferation tended to be higher for MSCs exposed to the uninjured spinal cord, no significant difference could be detected between the experimental groups. The proliferation rate of transplanted MSCs was about one ninth of the proliferation rate in culture (Additional file 4: Figure S4B1-B3). MSCs downregulated gene expression of Caspase-3 following transplantation into both injured (− 0.79) and uninjured spinal cord (− 0.72) (*p* < 0.001) (Fig. 4d). Histological examination revealed no relevant co-expression between mCherry+MSCs and Caspase-3 in the injured (0.4%, CI − 0.05–0.8%) and uninjured spinal cord (0.2%, CI − 0.2–0.7%) (Fig. 4e, h, i) using positive control as the reference (Additional file 6: Figure S6). No significant difference in the number of mCherry+Caspase-3+MSCs could be detected between MSCs in the injured and uninjured spinal cord (Fig. 4e). Gene expression analysis revealed an upregulation of TuJ1 in MSC[SCI] as compared to MSC[In vitro] (2.17, *p* < 0.001) while MSC[Naive] did not have an upregulation (0.66, *p* = 0.1) (Fig. 5b). However, MSCs upregulated gene expression of GFAP in both injured (4.78, *p* < 0.001) and uninjured spinal cord (5.69, *p* < 0.001). Histological examination of co-expression between mCherry and GFAP, NeuN, or TuJ1 revealed no double positive cells in neither uninjured (Fig. 5c, d) nor injured (Fig. 5c, e) spinal cord, suggesting a lack of detectable neuronal or astrocytic differentiation. Taken together, MSCs transplanted into the uninjured or injured spinal cord survive for 1 week and proliferate to a low extent but do not undergo neural or astrocytic differentiation.

## Discussion

In this study, we investigated the cellular response of MSCs following transplantation into spinal cord injury (SCI). We found that MSCs transplanted into SCI upregulate expression of CD45 and MHC-II and expression of genes related to cytokine production, phagocytosis/endocytosis, and immune cells/response and thereby adopt immune cell-like characteristics within the recipient.

Transplanted MSCs were detected in both injured and uninjured spinal cord at 7, but not 14, days following transplantation. The short-term survival is in line with the findings of Jung et al. [28] and others [13, 28–31]. However, it was not within the scope of this study to determine whether the fluorescence faded or if the MSCs perished. Considering that a commonly available expression construct was implemented, the fluorescence did not fade in culture conditions, and other authors have reported short-term survival, we confidently hypothesize that the MSCs perish between 7 and 14 days following transplantation. Hence, the lower limit of survival seems to be 7 days, but the upper limit remains uncertain. Even though the Caspase-3 expression in transplanted MSCs was negligible, the global transcriptional analysis revealed that transplanted MSCs significantly upregulated genes and GO/KEGG terms related to ubiquitination and apoptosis. In the few cells which were observed and reported as Caspase-3 positive, the staining was sometimes difficult to distinguish from phagosomes. However, considering that the global transcriptional analysis revealed an upregulation of genes related to phagocytosis, it is likely that the MSCs did indeed have phagosomes, which confirms their adoption of immune cell-like characteristics. Hence, the lack of detectable MSCs at 14 days post transplantation is likely cell death resulting from the challenging environment. Additionally, the downregulation of genes related to DNA repair in transplanted MSCs, in combination with the downregulation of genes related to cell-cycle activity, supports the theory that MSCs are struggling to survive within the recipient and simply perish between 7 and 14 days post transplantation. Given that the MSCs manage to secrete enough tissue and immunomodulatory factors [14, 15, 21, 32–40] during this time to enhance recovery, the short-term survival is not of great concern. However, it is interesting to speculate whether long-term survival could enhance recovery further. Transplanted MSCs did proliferate in both injured and uninjured spinal cord, but at about one ninth of the rate in culture. This is in line with the findings in the global transcriptional analysis which indicated that genes related to cell-cycle progression/regulation were downregulated and detected using EdU in a small number of cells. This in combination with the downregulation of genes related to DNA processes and repair indicates that the MSCs are active within the recipient but slowing down. The lower rate of proliferation in vivo as compared to in culture is most likely also a consequence of the hazardous SCI environment limiting the nutrition and oxygen supply and resulting in a less beneficial growth environment. Transplanted MSCs did not differentiate in the injured or uninjured spinal cord. Since MSCs are from mesodermal and not ectodermal origin, this result was expected. However, Aras et al. [4] and others found that transplanted MSCs could differentiate into astrocytes [12, 41, 42], oligodendrocytes [4, 43], and neurons [4, 6, 9]. Interestingly however, the global transcriptional analysis revealed that MSCs in SCI upregulated gene expression of TuJ1 and GFAP, which is more in line with previous reports. The lack of differentiation detected in this study might be due to the time point of evaluation, the tissue from which the MSCs were established, the severity of the injury, the type of injury, or a similar factor. However, most authors in the field are convinced that MSCs mediate their beneficial modulation of the microenvironment mainly by paracrine effects [2, 10, 13, 28]. Assuming that this is true, the lack of ectodermal differentiation is of minor importance. Taken together, MSCs survive for a brief period of time during which the MSCs do not undergo apoptosis but a fraction is active and proliferate. Moreover, MSCs do not undergo neural differentiation following transplantation, which supports the theory that MSCs act through tissue and immune system modulation early on following transplantation rather than trans-differentiation and integration.

Transplanted MSCs maintained their expression of CD29 and Sca1 but also expressed CD45 on the cell surface, which was in line with gene expression analysis. Hence, MSCs preserve stem cell characteristics but adopt an immune cell-like phenotype following transplantation. CD45 expression could indicate that the MSCs respond to the in vivo environment and therefore alter their phenotypic profile. The fact that the MSCs did express CD29 and Sca1 to a high extent indicates that the CD45 expression is not a result of the MSCs being phagocytized by macrophages. Transplanted MSCs maintained their expression of MHC-I but upregulated expression of MHC-II detected using flow cytometry, immunohistochemistry, and gene expression analysis. MSCs in culture conditions express MHC-II within the cell but not on the cell surface [27, 44]. Le Blanc et al. [27] demonstrated that MSCs in culture, exposed to IFNγ for 48 h, express MHC-II on the cell surface. IFNγ is critical for the innate and adaptive immune response and is present in a SCI environment [45, 46]. Thus, the ability of IFNγ to induce MHC-II expression seems to be true not only in vitro but also in vivo. Moreover, MHC-II is a macrophage marker which further supports that MSCs adopt an immune cell-like phenotype. The histological evaluation clearly demonstrated the MHC-II expression. Careful inspection of the MSCs reveal structures which might resemble phagosomes, which is in line with and supports the MHC-II expression considering that MSCs transform into macrophage-like cells and should gain the ability to phagocytize, which was also predicted by the global transcriptional analysis. The fact that MSCs maintain their CD29 and Sca1 expression significantly lowers the probability of the MSCs being engulfed by macrophages and rather supports the fact that the cells are still MSCs, but with an immune cell-like phenotype and function and they themselves phagocytize. This is further supported by the fact that the immune cells clearly surrounded the graft but did not enter into it as revealed by Additional file 5: Figure S5. Furthermore, global transcriptional analysis revealed that MSCs upregulate genes related to immune system response, phagocytosis/endocytosis, and cytokine production and release, which further supports the fact that MSCs seem to take an immune cell-like phenotype following transplantation. Genes related to both the innate and the adaptive immune system but also to leukocytes (CD45+ cells) were identified. The upregulation of genes related to production and release of pro-inflammatory cytokines further suggests that MSCs take a macrophage-like profile with secretory capabilities. Furthermore, this sheds new light on the assumed immune privilege of MSCs and might have to be taken into account in MSC-based therapies, not limited to SCI. Taken together, transplanted MSCs upregulate expression of CD45 and MHC-II and thereby adopt an immune cell-like phenotype but also upregulate genes related to cytokine production, immune cells/response, and phagocytosis/endocytosis indicating that the MSCs also undertake immune cell-like functions and that this is induced by the SCI environment.

Cytokine expression is usually upregulated in both the spinal cord and the cerebrospinal fluid following SCI as a result of activation of macrophages. We found that genes relating to cytokine stimulus response and production were upregulated in MSCs transplanted into SCI and that pathways related to TNFα were affected specifically. Both IL-1β [47] and TNFα [48] activate NF-κβ-dependent transcription [49]. The increased response to cytokines coincided with significant upregulation of genes related to signaling pathways, especially NF-κβ signaling pathways. MSCs upregulated GO terms related to increased response to IFNγ which might be related to the elevated MHC-II expression on MSCs, as indicated by Le Blanc et al. [27].

Extracellular vesicles are secreted from endosomal compartments of MSCs (MSC-EVs) [50, 51] and have been characterized as mediators of the immune-modulatory effects of MSCs [15, 32–40]. Assuming that MSCs mediate the majority of their therapeutic effect through paracrine effects, the content of the MSC-EVs is highly interesting to investigate. But, it is reasonable to hypothesize that at least some of the transcriptional changes induced in MSCs by the in vivo environment are transmitted to the surrounding tissue using MSC-EVs. Identifying, isolating, and sequencing MSC-EVs released by transplanted MSCs would potentially reveal the full mechanism of action of MSCs. Given that the cargo—or at least essential parts of the cargo—could be constructed synthetically and administrated directly to a patient, the need for MSC transplantation could perhaps be abolished. This study however aimed at investigating the cellular response of transplanted MSCs and did not aim to identify any potential therapeutic agents secreted by the MSCs.

The low number of genes significantly differentially expressed between MSC[Naive] and MSC[SCI] indicated a similarity between these two experimental groups. The comparison between these two and MSC[In vitro] indicated up- and downregulation of the same type of functional categories further emphasizing their similarity. Evaluation of phenotypes, proliferation, survival, and differentiation also indicated a striking similarity between MSC[SCI] and MSC[Naive]. The similarity between MSC[SCI] and MSC[Naive] is probably a consequence of the non-significant injury caused by the glass capillary needle during transplantation. This is not only a limitation in this study, but also a limitation in stem cell transplantation as a therapeutic approach for SCI. Furthermore, the results presented in this study might vary with the time point of evaluation, the severity of the injury, the injury type, the species, and/or the strain. One additional crucial limitation to keep in mind in all transplantation studies is transplantation accuracy. Furthermore, the results in this study were not adjusted for differences in force, displacement, and/or velocity of the SCI.

## Conclusion

In this study, we found that MSCs transplanted into SCI upregulate expression of CD45 and MHC-II and expression of genes related to cytokine production, phagocytosis/endocytosis, and immune cells/response and thereby adopt immune cell-like characteristics within the recipient. Understanding the cellular response of MSCs allows for modification, enhancement, and/or replacement of the effect mediated by MSCs. This could lead the way toward not only a more efficient therapy, but perhaps also a cell-free therapy that could be beneficial in terms of recovery, time, effort, and cost.

## Additional files

Additional file 1: **Figure S1.** MSCs pre- and post transplantation. **Figure S1**A, B mCherry+MSCs prior to transplantation (48 h following transfection). **Figure S1**C mCherry+MSCs transplanted, sorted (FACS) following 7 days in the recipient, plated and kept in culture for 72 h. Fluorescence of mCherry in MSCs evaluated using excitation at wavelength 633 nm. **Figure S1**D co-expression of mCherry and Sca1 in MSCs isolated from spinal cord 7 days following transplantation. (TIF 27481 kb)

Additional file 2: **Figure S2.** Physical parameters and gating strategy of transplanted MSCs. **Figure S**2A-C non-transplanted non-transfected MSCs. **Figure S**2D-F non-transplanted transfected MSCs. **Figure S**2G-I uninjured spinal cord. **Figure S**2J-L uninjured spinal cord with transplanted transfected MSCs. **Figure S**2M-O injured spinal cord. Figure S2P-R injured spinal cord with transplanted transfected MSCs. (TIF 46283 kb)

Additional file 3: **Figure S3.** Global transcriptional changes in MSCs - extended analysis. **Figure S3**A two first components/variables following dimensionality reduction using principal component analysis (PCA) and *t*-distributed stochastic neighbor embedding (tSNE, perplexity = 3, theta = 0.5). Each dot represents one biological replicate. Ellipse represents 95% confidence interval. **Figure S3**B bootstrapped (1000 runs) first three components of PCA clustered using affinity propagation (left) and k-means clustering (right, 3 clusters, 20 starts). **Figure S3**C two first components following PCA and tSNE clustered using K-means clustering (KM, 3 clusters, 20 starts), affinity propagation (AP), expectation maximum (EM), and K-nearest neighbor (KNN, 60:40 split). Figure S3D top positive and negative loadings for the first and second principal component following PCA. (TIF 23979 kb)

Additional file 4: **Figure S4.** Proliferation and expression of CD29, Sca1 and CD45 in non-transplanted MSCs. **Figure S4**A experimental design. **Figure S4**B1-B3 expression of EdU in mCherry+MSCs. **Figure S4**C1-C5 expression of CD29, Sca1, and CD45 in mCherry+MSCs. Figure S4C6-C8 co-expression of CD29, Sca1, and CD45 in mCherry+MSCs. (TIF 16399 kb)

Additional file 5: **Figure S5.** Interaction between transplanted MSCs and immune cells. MSCs in relation to immune cells at 7 days following transplantation into injured spinal cord. (TIF 6636 kb)

Additional file 6: **Figure S6.** Caspase-3 positive control. Mouse liver tissue stained with Caspase-3 acting as positive control to Caspase-3 staining of mCherry+MSCs. Scale bar represents 20 μm. (TIF 894 kb)

## Abbreviations

SCI: Spinal cord injury
MSC: Mesenchymal stem cell
MSC[Naive]: Mesenchymal stem cells transplanted into an uninjured spinal cord
MSC[SCI]: Mesenchymal stem cells transplanted into an injured spinal cord
MSC[In vitro]: Non-transplanted mesenchymal stem cells
ORA: Over-representation enrichment analysis
NTA: Network topology-based analysis
GO: Gene Ontology
KEGG: Kyoto Encyclopedia of Genes and Genomes
BP: Biological process (GO)
CC: Cellular component (GO)
MF: Molecular function (GO)
FDR: False discovery rate
LogFC: Log2(fold change)
Log2(CPM): Log2(counts per million)
EV: Extracellular vesicle
PCA: Principal component analysis
tSNE: *t*-distributed stochastic neighbor embedding
