# Supplementary figures and images for: Mesenchymal stem cells transplanted into spinal cord injury adopt immune cell-like characteristics
Source: Stem Cell Res Ther. 2019 Apr 3;10:115. doi: 10.1186/s13287-019-1218-9 (PMC6448247; doi:10.1186/s13287-019-1218-9)

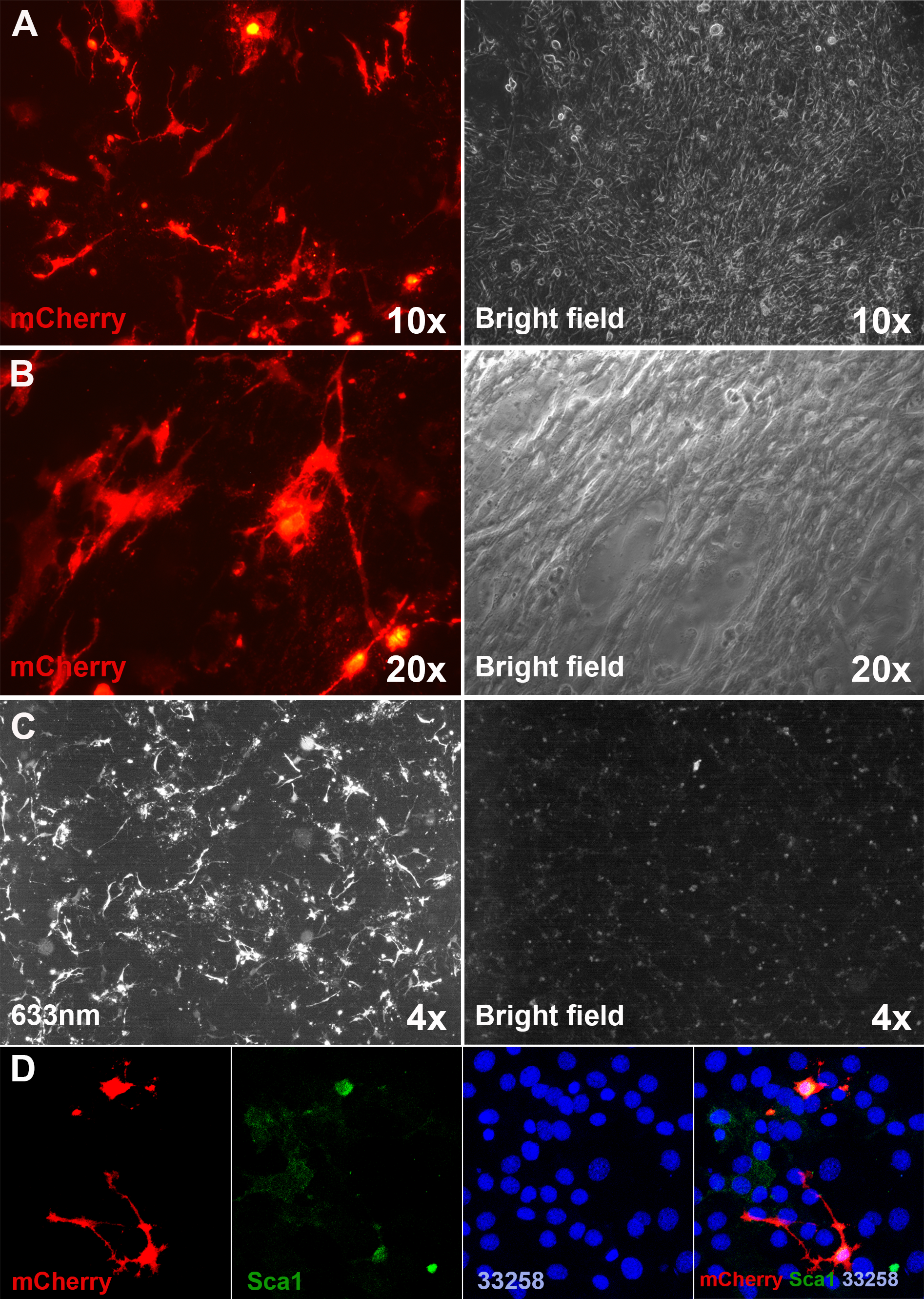

Supplement: Supplementary file 1 — Figure S1. MSCs pre- and post transplantation. Figure S1A, B mCherry+MSCs prior to transplantation (48 h following transfection). Figure S1C mCherry+MSCs transplanted, sorted (FACS) following 7 days in the recipient, plated and kept in culture for 72 h. Fluorescence of mCherry in MSCs evaluated using excitation at wavelength 633 nm. Figure S1D co-expression of mCherry and Sca1 in MSCs isolated from spinal cord 7 days following transplantation. (TIF 27481 kb) [file 13287_2019_1218_MOESM1_ESM.tif]

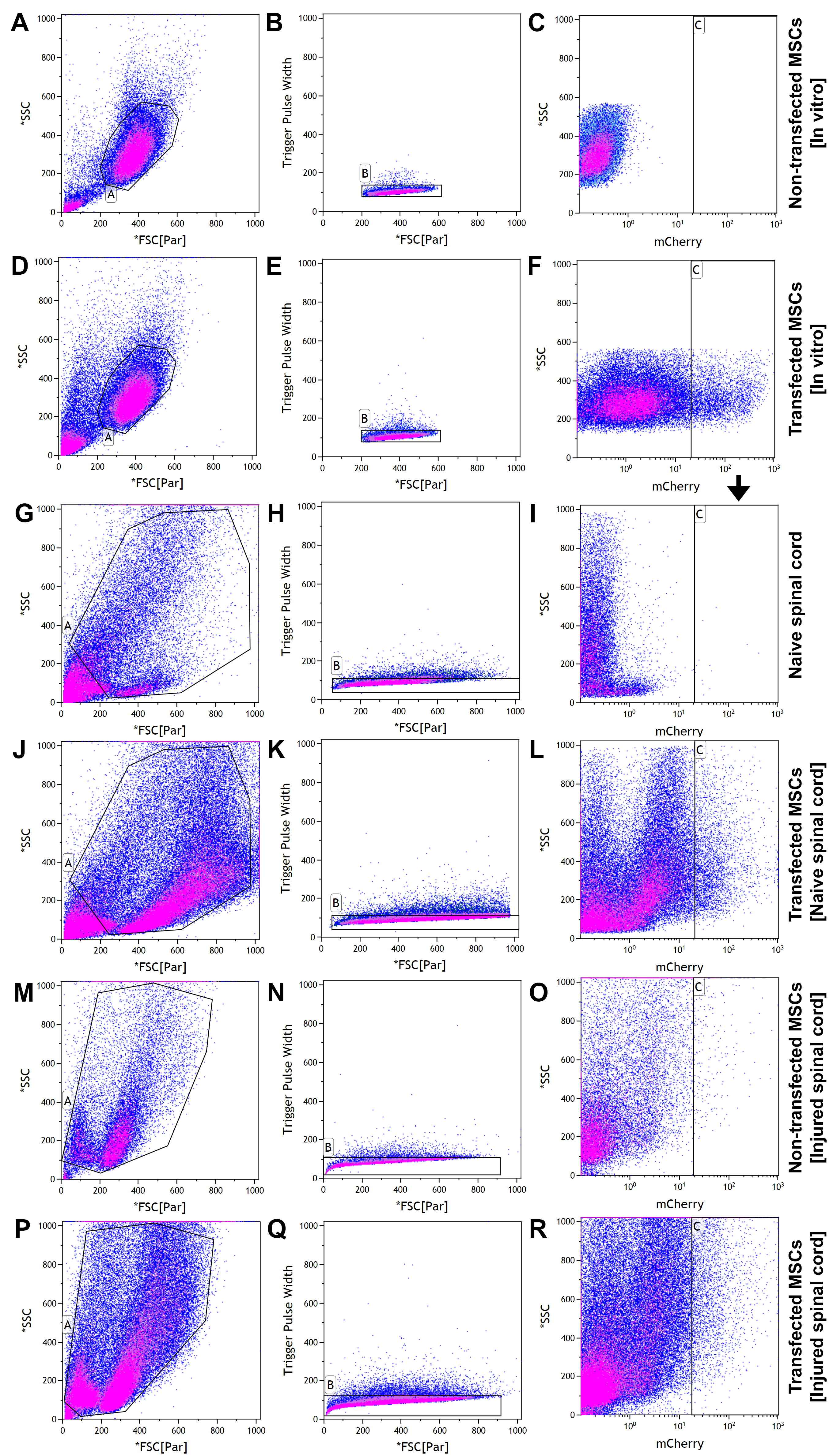

Supplement: Supplementary file 2 — Figure S2. Physical parameters and gating strategy of transplanted MSCs. Figure S2A-C non-transplanted non-transfected MSCs. Figure S2D-F non-transplanted transfected MSCs. Figure S2G-I uninjured spinal cord. Figure S2J-L uninjured spinal cord with transplanted transfected MSCs. Figure S2M-O injured spinal cord. Figure S2P-R injured spinal cord with transplanted transfected MSCs. (TIF 46283 kb) [file 13287_2019_1218_MOESM2_ESM.tif]

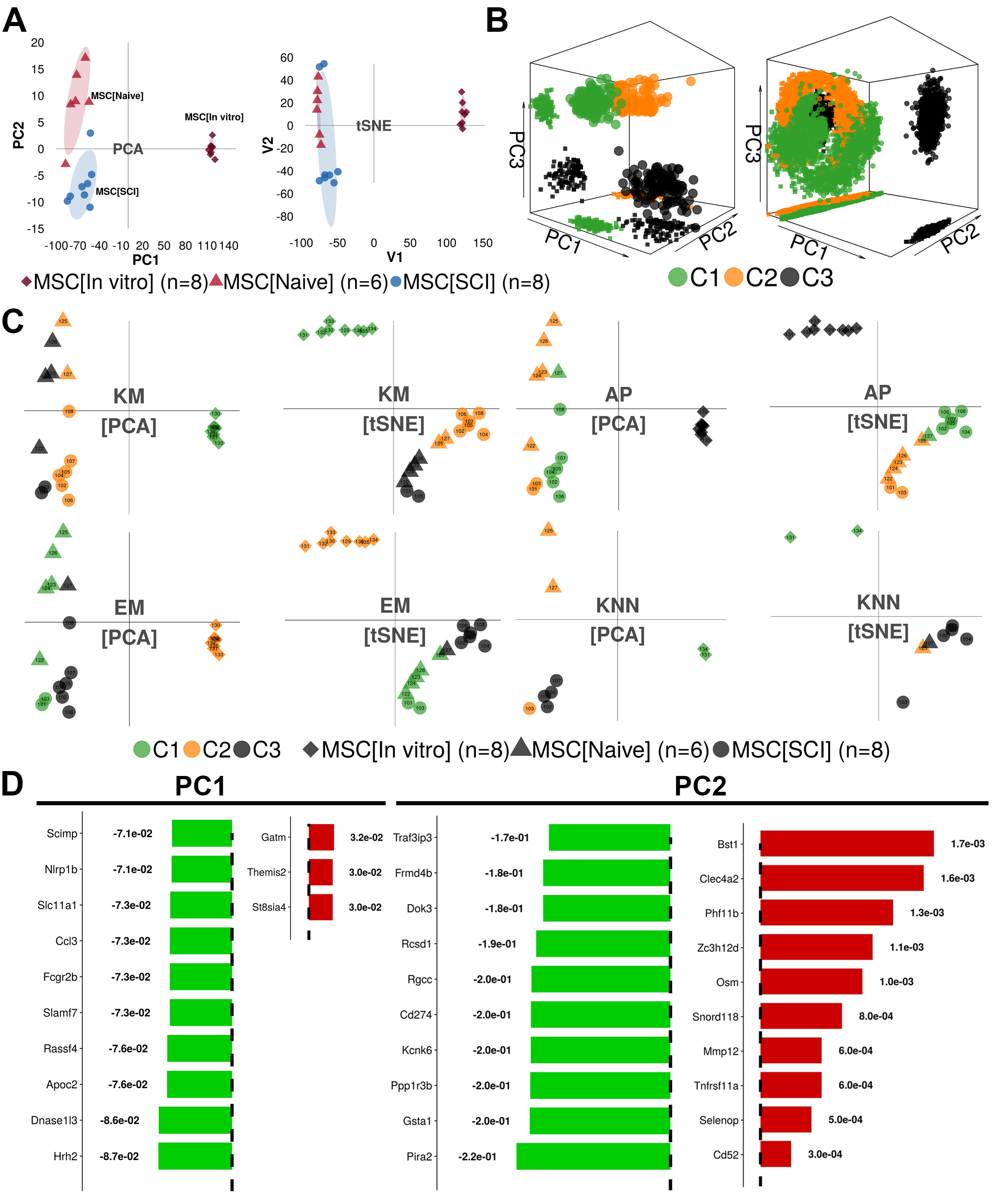

Supplement: Supplementary file 3 — Figure S3. Global transcriptional changes in MSCs - extended analysis. Figure S3A two first components/variables following dimensionality reduction using principal component analysis (PCA) and t-distributed stochastic neighbor embedding (tSNE, perplexity = 3, theta = 0.5). Each dot represents one biological replicate. Ellipse represents 95% confidence interval. Figure S3B bootstrapped (1000 runs) first three components of PCA clustered using affinity propagation (left) and k-means clustering (right, 3 clusters, 20 starts). Figure S3C two first components following PCA and tSNE clustered using K-means clustering (KM, 3 clusters, 20 starts), affinity propagation (AP), expectation maximum (EM), and K-nearest neighbor (KNN, 60:40 split). Figure S3D top positive and negative loadings for the first and second principal component following PCA. (TIF 23979 kb) [file 13287_2019_1218_MOESM3_ESM.tif]

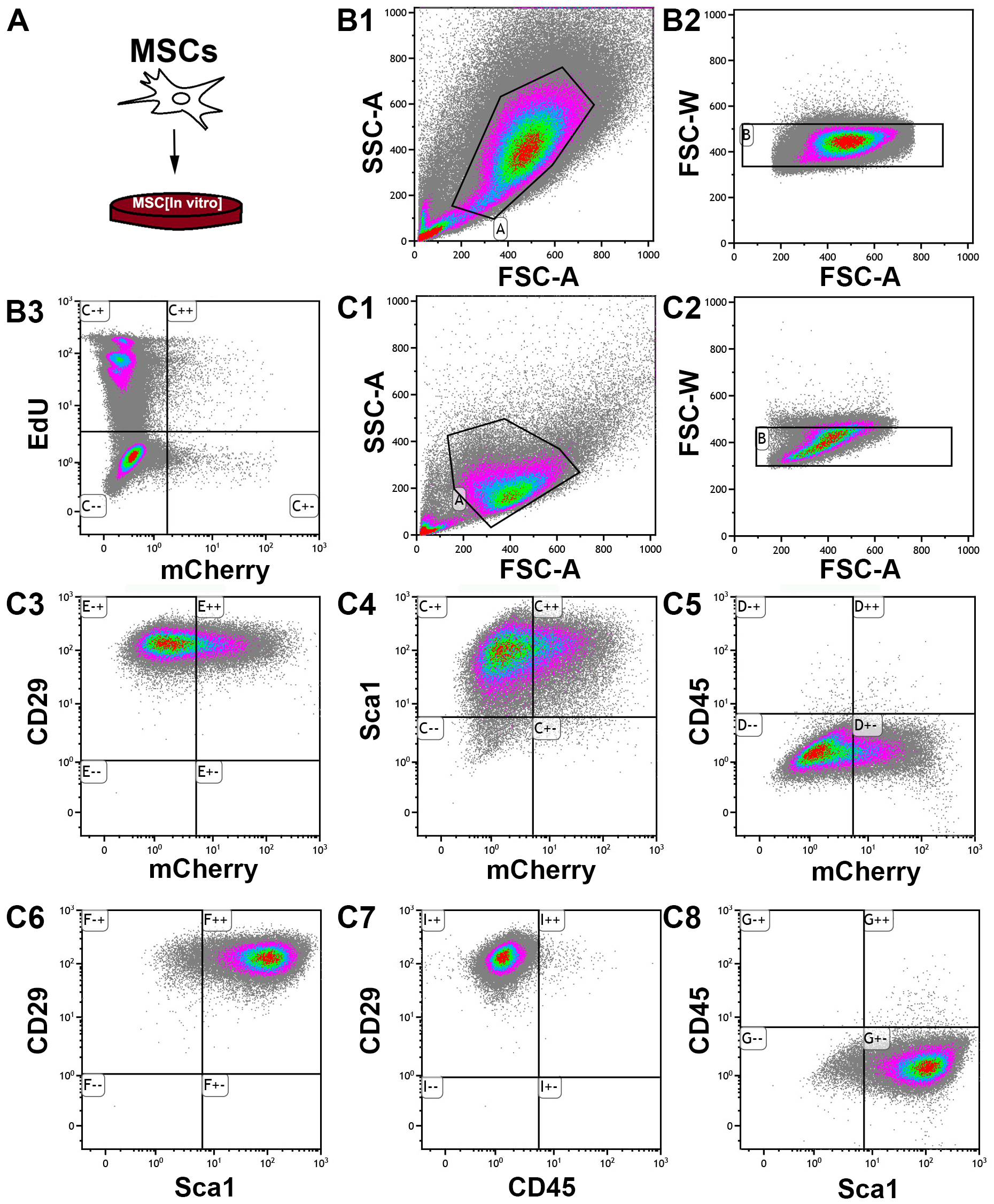

Supplement: Supplementary file 4 — Figure S4. Proliferation and expression of CD29, Sca1 and CD45 in non-transplanted MSCs. Figure S4A experimental design. Figure S4B1-B3 expression of EdU in mCherry+MSCs. Figure S4C1-C5 expression of CD29, Sca1, and CD45 in mCherry+MSCs. Figure S4C6-C8 co-expression of CD29, Sca1, and CD45 in mCherry+MSCs. (TIF 16399 kb) [file 13287_2019_1218_MOESM4_ESM.tif]

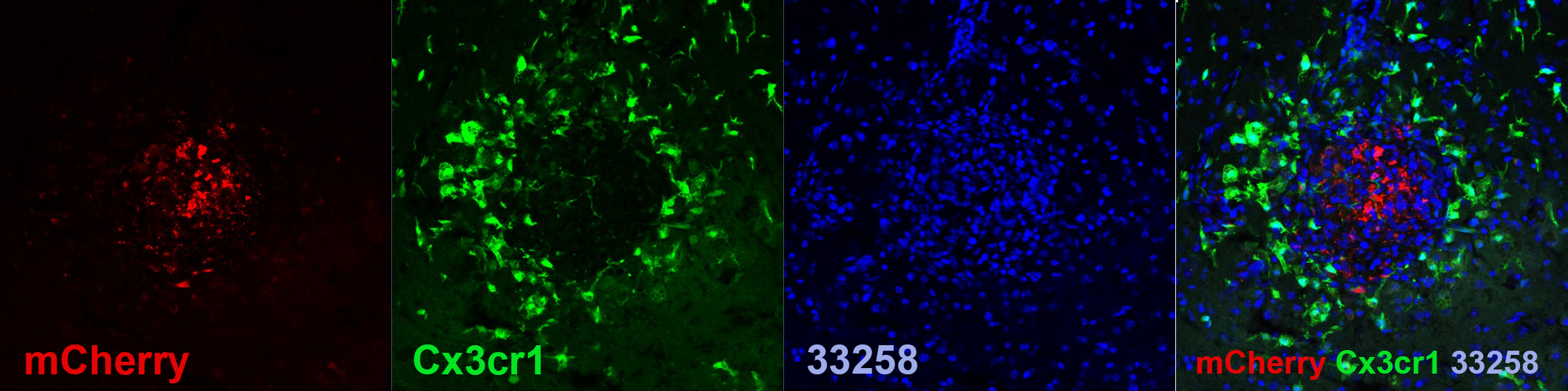

Supplement: Supplementary file 5 — Figure S5. Interaction between transplanted MSCs and immune cells. MSCs in relation to immune cells at 7 days following transplantation into injured spinal cord. (TIF 6636 kb) [file 13287_2019_1218_MOESM5_ESM.tif]

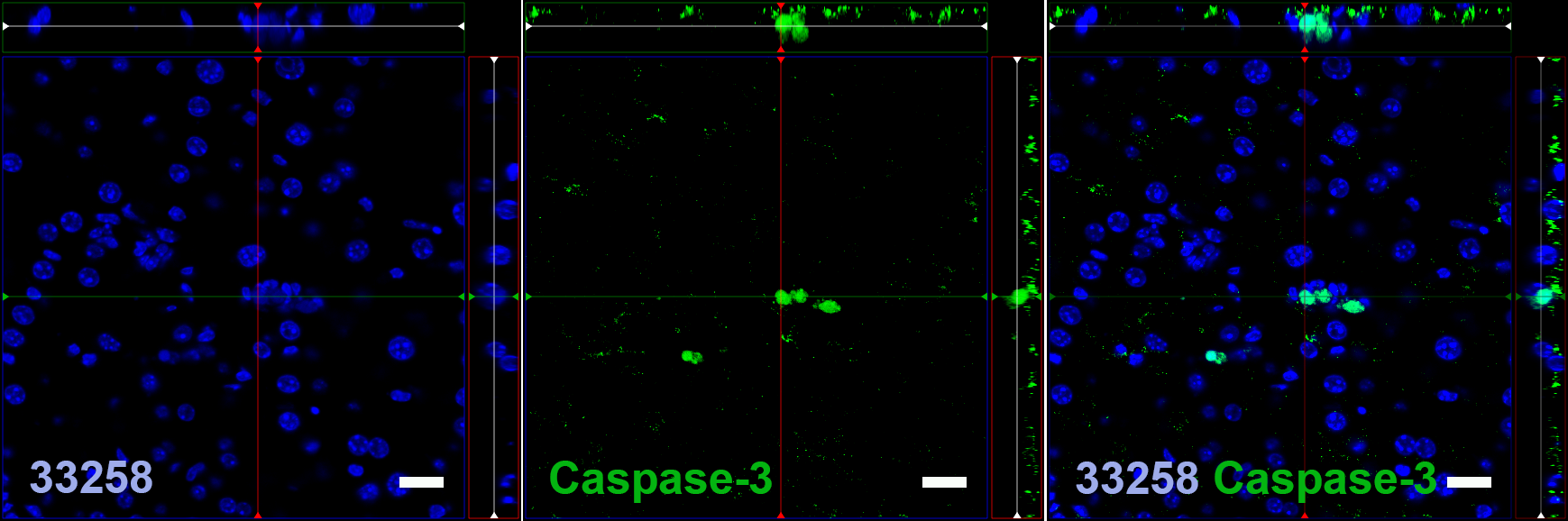

Supplement: Supplementary file 6 — Figure S6. Caspase-3 positive control. Mouse liver tissue stained with Caspase-3 acting as positive control to Caspase-3 staining of mCherry+MSCs. Scale bar represents 20 μm. (TIF 894 kb) [file 13287_2019_1218_MOESM6_ESM.tif]
